# Association of COVID-19 infection with large thrombi in left and right atrial appendages

**DOI:** 10.1186/s43044-021-00207-z

**Published:** 2021-09-16

**Authors:** Saeed Ghodsi, Sara Taghi, Zahra Alizadeh-Sani, Yaser Jenab, Zahra Hosseini, Laura Vaskelyte

**Affiliations:** 1 Department of Cardiology, Tehran Heart Center, Tehran University of Medical Sciences, North Kargar Street, 14111713138 Tehran, Iran; 2 Rajaie Cardiovascular Medical and Research Center, Iran University of Medical Sciences, Tehran, Iran; 3 Omid Hospital, Iran University of Medical Sciences, Tehran, Iran; 4 Tehran University of Medical Sciences, Tehran, Iran; 5 CardioVascular Center, Frankfurt, Germany

**Keywords:** COVID-19, Inflammation, Thrombus, Atrial fibrillation, Hypertrophic cardiomyopathy, Atrial appendage

## Abstract

**Background:**

Multiple intra-atrial thrombi are found rarely except in the presence of prosthetic valves, intra-cardiac devices, structural connections like foramen ovale and thrombophilia.

**Case presentation:**

We reported acute thrombosis formation in right and left atrial appendages of a 66-year old man admitted due to progressive dyspnea since 7 days earlier. He had a history of prior laryngeal Squamous Cell Carcinoma, apical hypertrophic cardiomyopathy (HCM), and atrial fibrillation (AF). Infection with COVID-19 was confirmed thereafter. Cardiac Magnetic Resonance Imaging (CMR) suggested the diagnosis of atrial clot superior to neoplasm. After surgical removal of the thrombi, symptoms as well as imaging features of pneumonia were resolved.

**Conclusions:**

We should focus on different presentations and complications of systemic inflammation especially in the setting of COVID-19 infection. Although risk factors of thrombosis are present in some of these patients, rapid progression as well as unusual types of involvement may indicate to a new trigger.

## Background

During the current pandemic of Corona Virus disease (COVID-19) [1], emerging evidence aggregated regarding its different features and associated disorders. Pulmonary and systemic inflammation occurred in this viral infection in addition to a prothrombotic state may potentially contribute to or amplify the rate of clot formation. Although finding a left atrial appendage thrombus is mainly linked to atrial fibrillation (AF), no considerable association with thrombophilia have been described [2]. However, co-occurrence of new large thrombi in both right and left atria in the absence of patent foramen ovale is uncommon.

## Case presentation

A 66 year-old man was admitted in emergency department of Tehran Heart Centre with recent exacerbation of progressive dyspnea aggravated as NYHA class II to III within  a week earlier, malaise, dizziness, and perspiration. He was a known case of apical hypertrophic cardiomyopathy (HCM), persistent atrial fibrillation, and laryngeal squamous cell carcinoma diagnosed since 5 month earlier. He had received two courses of chemotherapy schedule followed by a regular period of local radiotherapy for 30 successive sessions. The patient had underwent coronary angiography, 8 months prior to admission which determined the presence of mild non-obstructive coronary artery disease. He had a history of heavy cigarette smoking as well as opium addiction. He also mentioned relatively regular use of Warfarin over 6 months prior to admission. Other medications prescribed earlier included furosemide 20 mg once a day, ASA 100 mg daily, and atorvastatin 20 mg once daily.

Initial vital signs revealed an irregularly irregular pulse (heart rate: 65 bpm), tachypnea (respiratory rate: 21/min), hypoxemia (pulse oxygen saturation: 88%), and low-grade fever recorded as 38.1 C^0^. Recorded blood pressure was 100/65 mmHg. The patient’s laboratory data on admission were as following:

WBC: 5000/mm^3^ (with 26.8% lymphocytes), haemoglobin: 13.1 gr/dl, platelet: 168,000/mm^3^, C-Reactive Protein (CRP):11.4 mg/L, INR: 2.43, creatinine: 1.3 mg/dl, magneseium: 1.5 meq/L, K: 4.2 meq/L.

**Fig. 1:**
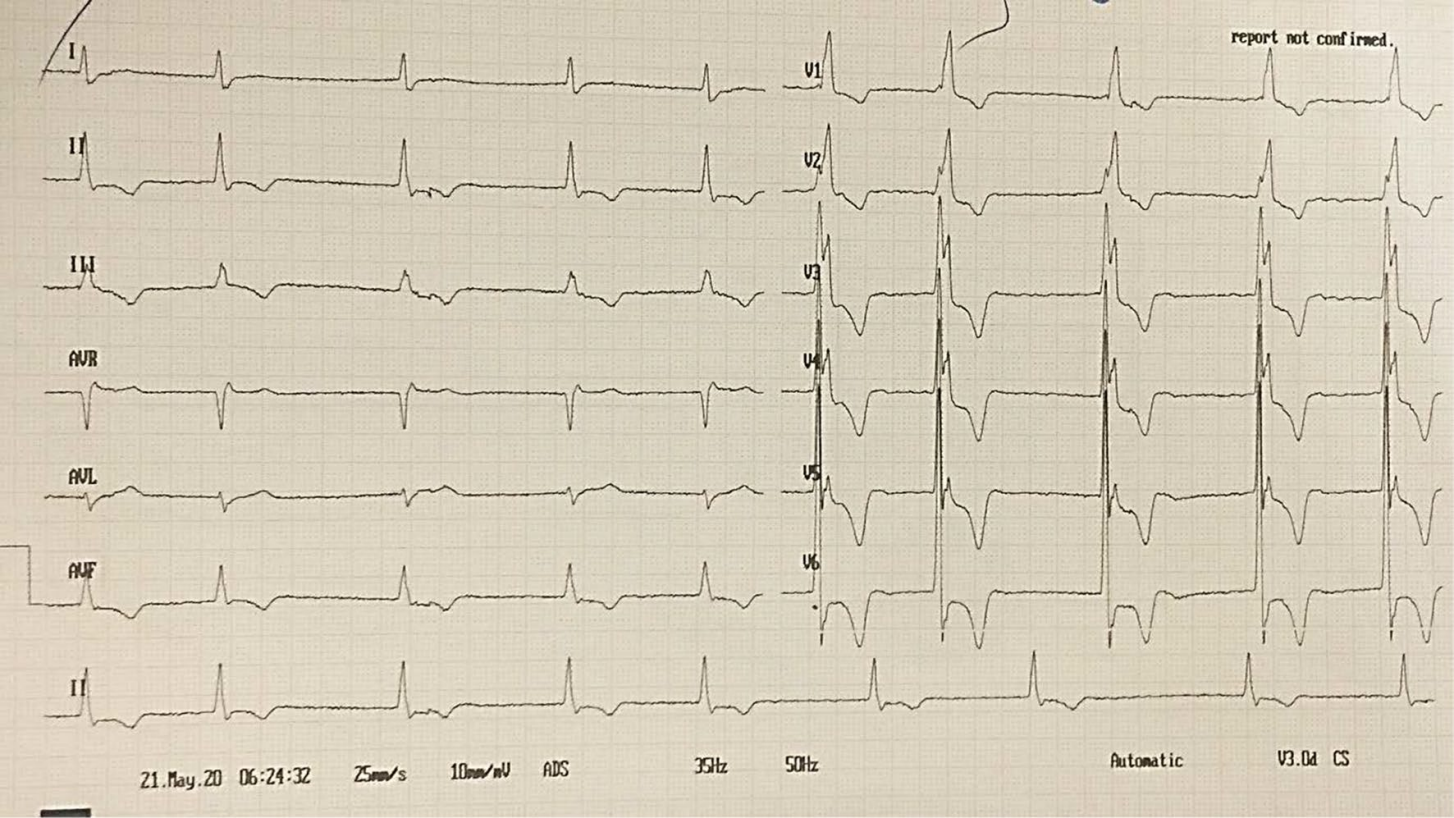
This figure depicts the 12-lead ECG of the patient obtained at the emergency department. Underlying rhythm is atrial fibrillation with normal axis, right bundle branch block, positive voltage criteria for left ventricular hypertrophy, and widespread deep T-wave inversion markedly seen in precordial leads in favour of hypertrophic cardiomyopathy

A 12-lead electrocardiogram was obtained which showed the rhythm of atrial fibrillation accompanied with right bundle branch block, voltage criteria pertaining to left ventricular hypertrophy (LVH) denoting HCM, and non-specific ST depression with giant inverted T waves in precordial as well as in inferior leads (Fig. 1).

**Fig. 2:**
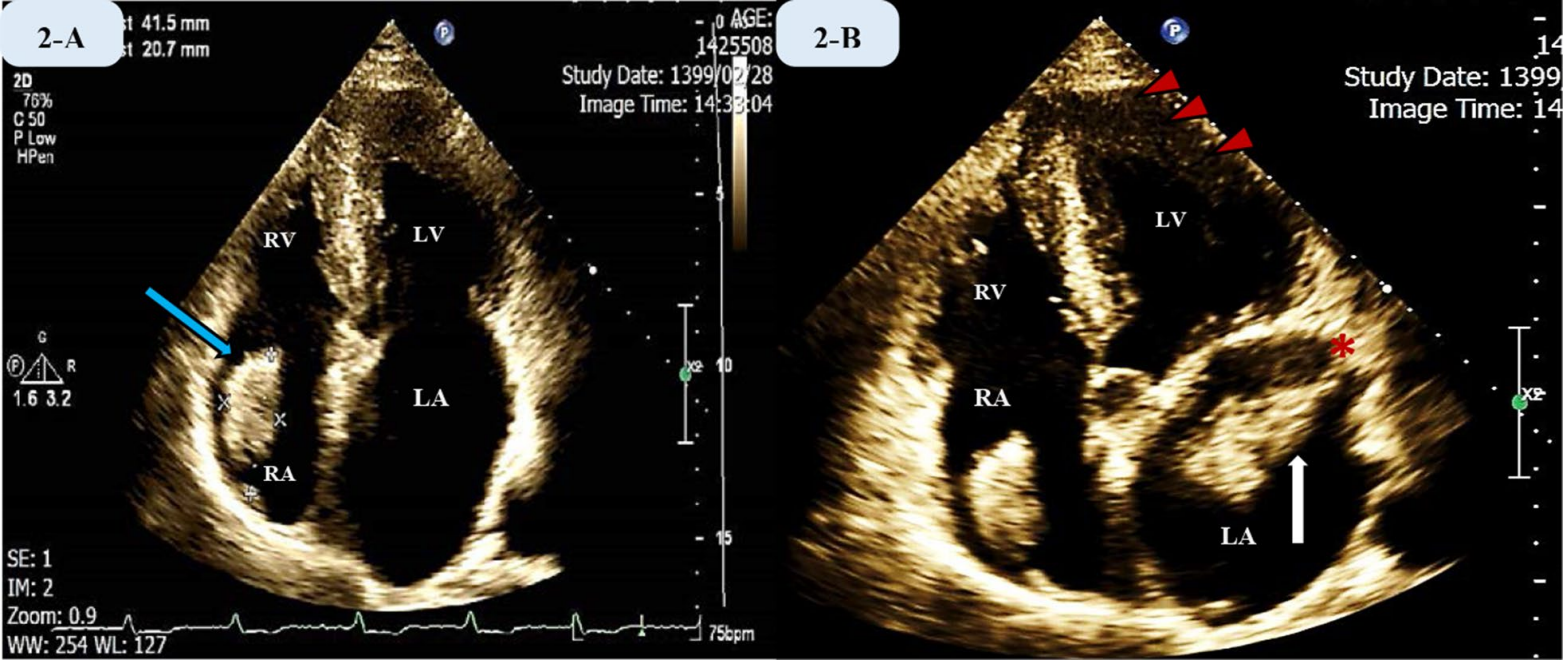
Transthoracic echocardiographic images showing two large atrial masses as well as extreme LA dilatation. **A** Apical four-chamber view illustrating a large pedunculated homogenous, well-demarcated mass with 42 mm*21 mm dimensions attached to right atrial appendage (indicated via a blue oblique arrow). **B** Apical five-chamber view showing concomitant presence of two atrial masses. The vertical white arrow points to a large lobulated mass in LA with well-defined borders attached to LAA (red asterisk) with a stalk. Red arrowheads depict the apical to mid ventricular extension of hypertrophy. LV: left ventricle, RV: right ventricle, LA: left atrium, RA: right atrium

Transthoracic echocardiography demonstrated normal size and function of the left ventricle. Left ventricular ejection fraction (LVEF) was 60% with evident hypertrophy of all apical segments extending to mid part of the ventricle. Normal right ventricle (RV) size with moderate RV dysfunction were observed. Both atria were enlarged severely as measured by indexed volumes that were 65 ml/m^2^ and 57 ml/m^2^, respectively. There were also two large, fixed, wide based homogenous masses originating from left and right atria. Corresponding dimensions for the left atrial appendage mass and the other one attached to right atrial appendage were (50 × 24 mm) and (42 × 20 mm) respectively. Pulmonary artery pressure was found within normal range (PAP = 23 mmHg). Figure 2 illustrates the morphology and anatomic locations of the atrial masses.

**Fig. 3:**
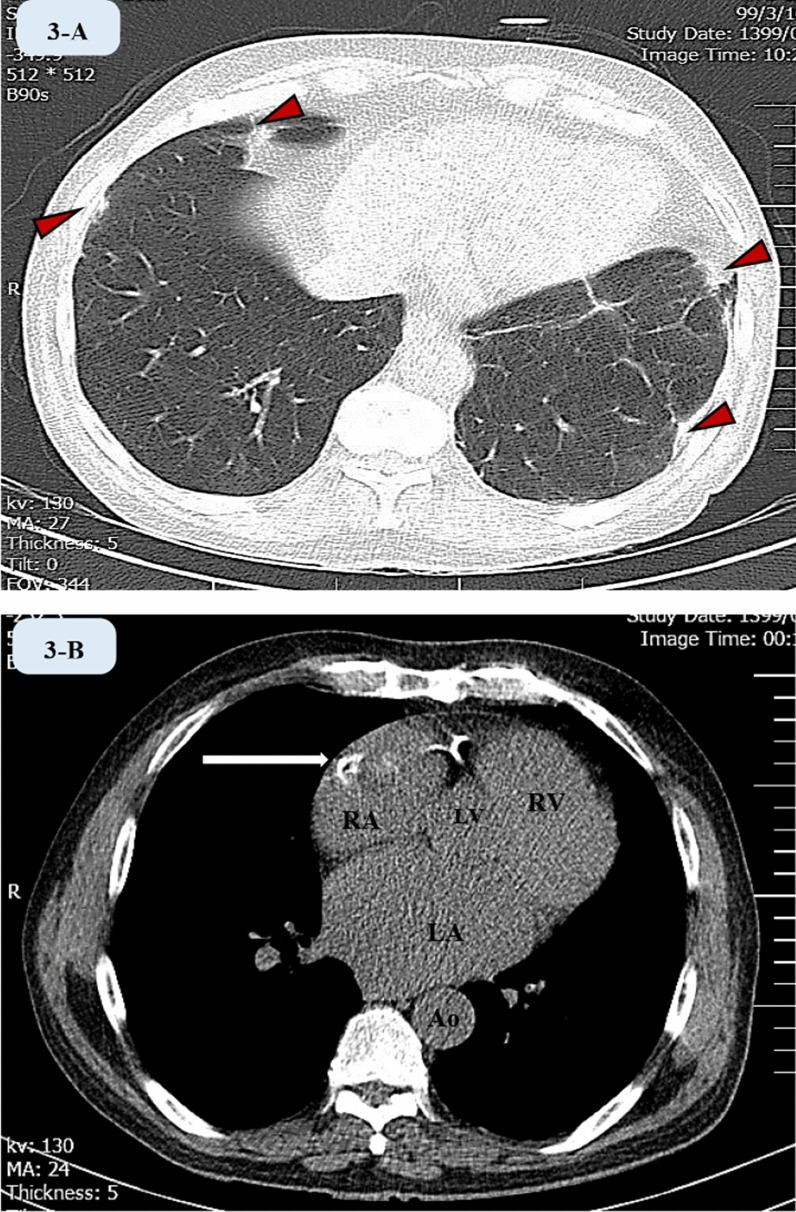
Computed tomography images.**A** Cross-sectional image of parenchymal window of the lungs showing bilateral fibrosis and mosaic pattern in both lungs with peripheral patchy ill-defined ground glass densities in favour of COVID-19 pneumonia (depicted via arrowheads). **B** Cross-sectional CT scan of the heart and mediastinum at the level of atria showing biatrial enlargement and a calcified space-occupying mass in RA (arrow). CT: computed tomography, LV: left ventricle, RV: right ventricle, LA: left atrium, RA: right atrium, Ao: descending aorta

A chest CT scan was performed which showed bilateral apical fibrosis in both lungs with peripheral patchy ill-defined ground glass densities in favour of COVID-19 pneumonia (Fig. 3).

The result of initial polymerase chain reaction (PCR) test for COVID-19 was positive. Thus, the patient received supportive medical care such as hydration with normal saline, non-invasive positive pressure ventilation (NIPPV), and hemodynamic monitoring. Intravenous unfractionated heparin was started immediately and he was treated with Hydroxychloroquine to reduce inflammation associated with COVID-19. Subsequent lab tests obtained during first week revealed elevated inflammatory markers. Increased levels of CRP, D-dimer and relative leukocytosis were detected. Values pertaining to CRP, D-dimer, and WBC were 14.6 mg/L, 2250 ng/ml, and 11,250, respectively.

**Fig. 4:**
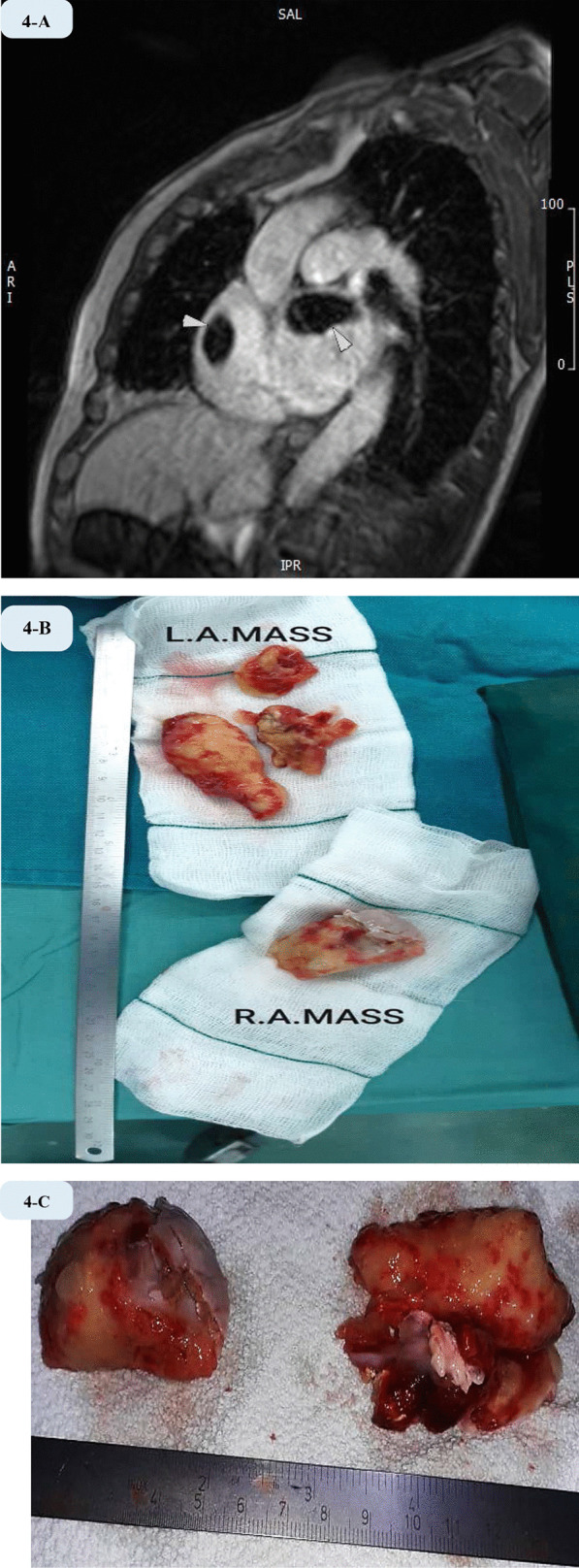
Cardiac MRI and gross image of the large atrial masses. **A** Short-axis inversion recovery early-enhancement image obtained with inversion time of 600 ms and demonstrated two large clots within RAA and LAA (arrowheads) which are low signal (dark) on the image. **B** This montage shows the two atrial masses after surgical resection. **C** This image illustrates the main body of the two masses

Cardiac magnetic resonance imaging (CMR) revealed two large masses in left and right atrial appendages suggestive for thrombi (Fig. 4A).

A flare up episode of the disease occurred in the second week. However the inflammatory situation was managed conservatively without intubation. Then, symptoms, oxygen saturation and hemodynamic parameters improved gradually. Over the third week, a clinical steady state was achieved. After 14 days of treatment, COVID -19 PCR and chest CT scan were repeated. Although second PCR of the specimen via nasopharyngeal swab was negative but CT scan still showed residual parenchymal involvement of the lungs. Therefore, surgical operation was deferred about 35 days since initial visit. Since size of the clot did not change over a period of one month under stringent anticoagulation, and subsequent thromboembolism was likely, surgical removal was planned. Furthermore, watchful waiting in this situation is controversial and there is not enough high-quality evidence to support close observation or treatment of choice. Finally, the patient underwent open cardiac surgery, which resulted in excision of the two large atrial masses (Fig. 4B, C). Pathologic examination reported the presence of organized thrombus in combination with fibrosis, and interstitial tissue. The left atrial mass consisted of three pieces of grey-brownish elastic tissue totally measuring 60*50*20 mm, that were attached to each other. Further sectioning of both masses showed areas of yellowish discoloration and heterogeneous surfaces. The right sided mass appeared as a piece of cream-greyish elastic tissue with diameters of 50*25*20 mm. The patient had considerable recovery of signs, hemodynamic measures, while his symptoms improved significantly, until he was discharged 10 days after surgery. Total hospital stay was 45 days. In the first follow-up visit arranged 1 month after discharge, he seemed completely recovered. However, his functional capacity was still suboptimal and mild residual weakness was noticed. Second visit was set for 45 days later. All symptoms including scarce coughs finally disappeared.

## Discussion

Atrial fibrillation contributes to a four- to five-fold increased risk of ischemic stroke and other thromboembolic events [3]. Prior investigations have declared that the residual risk of thromboembolism despite adequate anticoagulation remains considerable in about 30% of patients [4]. Thrombus formation in studies using transesophageal echocardiography (TEE) have demonstrated that left atrial thrombus particularly in the presence of underlying HCM complicates the course of AF [5]. Although the exact mechanism of thrombus formation is unclear in this patient, multiple predisposing factors might be addressed. Hypertrophic cardiomyopathy, AF rhythm, underlying malignancy, chemo radiation,and blood stasis secondary to COVID-19 were expected to contribute in the process.However, development of multiple large clots as observed in our patient requires either a prolonged trajectory or acceleration of pathophysiologic mechanisms such as hypercoagulable states. Furthermore, simultaneous constitution of organized thrombi in both atrial appendages occurs as an uncommon presentation. The incidence of thrombus formation in LAA in the presence of predisposing factors such as AF rhythm is about 10–15%. Conversely, constitution of an organized RAA clot is almost rare with a frequency of less than 2%. [6, 7].

Herein, no signs of atrial clot were observed in recent follow up echocardiography performed 2 months earlier. Besides, the patient was asymptomatic until he experienced progressive exertional dyspnea since approximately 7 days prior to admission. Thus, a subacute disorder might have triggered or accentuated the establishment of two atrial thrombi. The patient was in the remission phase of laryngeal cancer.He had not received chemotherapy, and radiotherapy since 5 months earlier. Given this background, systemic inflammation appears to be a presumed common pathway. According to high clinical suspicion during the ongoing pandemic of COVID-19, thoracic CT scan as well as PCR assay confirmed the presence of corona virus pneumonia. A plausible hypothesis is the participation of inflammatory response associated with this infection in atrial clot formation [8]. Hence, pro-inflammatory cytokines in COVID-19 may mediate thrombosis with or without diffuse intravascular coagulation (DIC) [9]. Coagulation factors, generalized inflammation, and platelet activation may regulate the severity of host immune response mainly rendered as acute respiratory distress syndrome (ARDS) [10, 11]. Therefore, inflammatory storm, coagulation cascade, and amplified immune response perpetuate each other through potential interplays.In this setting, virus-mediated endothelial dysfunction, increased blood viscosity might also occur particularly in severe disease.

Tumor thrombosis has been also described as a potential etiology of atrial clot formation [12]. Extension of tumoral cells is usually seen in renal cell carcinoma, hepatocellular carcinoma or advanced lung cancers. However, this manifestation is unlikely in our patient with prior laryngeal squamous cell carcinoma in apparent remission [13]. In addition, the tumor was suppressed and no relapse was detected within last 5 months.

Opium use is also recognized as a potential risk factor for thrombosis via increasing plasma fibrinogen, plasminogen activator-inhibitor, and pro-inflammatory cytokines [14]. We did not explore the presence of different thrombophilia traits especially ATPIII deficiency which might explain thrombus formation despite optimal INR.

Rapid development of concomitant large clots in LAA and RAA is strikingly rare in the absence of remarkable structural heart disease, implantable devices, or a communication between the atria. Nevertheless, it has remained unclear whether these thrombi were innocent bystanders or participated in the recent crescendo-type symptoms. However, due to increased risk of systemic and pulmonary embolism, prompt diagnosis and treatment was necessary. Thus, CMR was performed to discriminate the likely diagnosis of thrombus from a probable neoplastic tissue. Moreover, anticoagulation with unfractionated heparin (UFH) was provided until surgery was done. Recent studies have found a 24.2% decrease in short-term (28-day) mortality of severe COVID-19 patients who had received sufficient thrombo-prophylaxis via low molecular weight heparin (LMWH) or UFH [15]. Furthermore, LMWH and UFH exhibit modest anti-inflammatory and immunomodulatory effects in addition to their anticoagulant function. These medications are able to stabilize endothelial cell glycocalyx in a dose dependent manner, which in turn, modifies leukocyte adhesion [16].

## Conclusions

There are many risk factors, which potentially trigger, precipitate, or augment thrombosis in a susceptible patient. Atrial fibrillation, Hypertrophic cardiomyopathy and prior laryngeal squamous cell carcinoma are underlying substrates for activated coagulation in our patient. However, acute formation of multiple large biatrial clots in a relatively short timeline indicates to a recent stimulating agent. In the present scenario, severe inflammation afforded via COVID-19 is thought to be the major culprit.

## Data Availability

Not applicable**. “**Data sharing not applicable to this article as no datasets were generated or analyzed during the current study.

## Abbreviations

COVID-19: Corona Virus Disease 2019
HCM: Hypertrophic cardiomyopathy
AF: Atrial fibrillation
CMR: Cardiac Magnetic Resonance Imaging
NYHA: New York heart association
LVH: Left ventricular hypertrophy
WBC: White Blood Cell
CRP: C-reactive protein
INR: International normalized ratio
UFH: Unfractionated heparin
LMWH: Low molecular weight heparin
LVEF: Left ventricular ejection fraction
RV: Right ventricle
CT: Computerized Tomography
PCR: Polymerase chain reaction
NIPPV: Non-invasive positive pressure
TEE: Transesophagial echocardiography
DIC: Diffuse intravascular coagulation
ARDS: Acute respiratory distress syndrome
